# The role of individual variables as antecedents of entrepreneurship processes: Emotional intelligence and self-efficacy

**DOI:** 10.3389/fpsyg.2022.978313

**Published:** 2022-10-25

**Authors:** María Inmaculada López-Núñez, Susana Rubio-Valdehita, Eva M. Díaz-Ramiro

**Affiliations:** Faculty of Psychology, Complutense University of Madrid, Madrid, Spain

**Keywords:** entrepreneurial intention, emotional intelligence, self-efficacy, personality, individual differences

## Abstract

Currently, entrepreneurship is a priority for economic, social, and technological growth. Therefore, the interest in understanding entrepreneurship processes has increased significantly. Individual variables play a fundamental role, and academic research has pointed out the influence of emotional intelligence in entrepreneurial processes; however, its relationship with other interpersonal processes and individual variables, such as personality and self-efficacy, has not been extensively studied. The aim of this research was to analyze the relationship among emotional intelligence, self-efficacy, and entrepreneurial intention, controlling for the effects of personality, gender, and age. Multiple hierarchical regression analyses were applied through a questionnaire survey of 1,593 college students to test the relationship between the constructs in the model. The results show that the personality traits are associated with entrepreneurial self-efficacy, emotional intelligence positively influences entrepreneurial intention, and self-efficacy mediates the relationship between emotional intelligence and entrepreneurial intention. Practical implications for training programs are examined, and future lines of research were discussed.

## Introduction

Entrepreneurship is one of the main goals to support society’s progress and to improve citizens’ employability, innovation, and economic growth. According to Leutner et al. (2014), entrepreneurship can be described as behaviors that create value by taking advantage of opportunities in an innovative and new way. Not only entrepreneurship is the creation of companies, but entrepreneurial behavior also implies discovering ideas and opportunities and carrying them out (Shane and Venkataraman, 2000).

The literature on entrepreneurship shows that it is a multidimensional behavior, a process rather than an isolated event, which results from planned activities, random events, individual variables, and social norms (Leyden and Link, 2015; Dimov, 2020). It is these complex interactions that lead more and more academics to talk about entrepreneurship processes (Brixy et al., 2012; McMullen and Dimov, 2013).

The interest in fostering entrepreneurial intention to promote innovation, economic growth and combat unemployment has led to extensive efforts to identify potential entrepreneurs, develop training for entrepreneurship, and identify key aspects in entrepreneurial processes (Sánchez, 2013). University students are particularly important in the research on entrepreneurial intention (Krueger et al., 2000; Barba-Sánchez et al., 2022). Research shows that entrepreneurs and students with high entrepreneurial intention have a similar psychological profile, which is characterized by high scores on extraversion, conscientiousness, openness, emotional intelligence, self-confidence, and ambiguity tolerance and low scores on agreeableness and neuroticism. This profile can predict entrepreneurial intention with a significant level of accuracy (López-Núñez et al., 2020).

Since behavior is based on individual differences, it can be assumed that these differences influence entrepreneurial intentions and behavior, regardless of whether the person is an employee, a self-employed worker, or a student (Ahmetoglu et al., 2011).

Entrepreneurial intention is the strongest predictor of entrepreneurial behavior. So that exploring the mechanisms that underlie the effect of individual variables on entrepreneurial intention will contribute to better understanding of the entrepreneurship process. Several scholars have examined widely the association between personality traits and entrepreneurial intention (Fellnhofer, 2018; Hu et al., 2018). Other studies have focused on the effect of emotional intelligence (EI) on entrepreneurial intention (Ingram et al., 2019) although there are fewer studies that have analyzed the relationship between EI and individual differences in entrepreneurship. While other studies, based on social cognitive theory, have provided empirical evidence that entrepreneurial self-efficacy (ESE) is a key cognitive predictor of entrepreneurial intention (Hu and Ye, 2017). Despite that, few studies have explored the role of entrepreneurial self-efficacy related to EI and entrepreneurial intention (Mortan et al., 2014; Chien-Chi et al., 2020).

Identifying the individual factors that predict entrepreneurial intention has great theoretical and practical significance. On the one hand, it will provide theoretical explanation for the relationship among EI, ESE, and entrepreneurial intention for a better understanding of the individual variables as antecedents of entrepreneurship process (Dimov, 2020; Matricano, 2020). On the other hand, the results may help to identify potential entrepreneurs and may be used to design more effective training strategies to develop the skills and competencies that both novice and emerging entrepreneurs need to face the challenges of their new projects and achieve success. (Cope, 2011) provides suggestions for the long-term development of entrepreneurship education.

This study was designed from the revision of the Theory of Planned Behavior (TPB; Ajzen, 2011), the social cognitive theory (SCT; Bandura, 2001), and the basic intention-based progress model (Krueger and Brazeal, 1994; Wu et al., 2018). According to TPB, entrepreneurial intention is influenced by personal attitudes (positive or negative evaluation about the intended behavior), subjective norms (the perceived social support to fulfill the intended behavior), and perceived behavioral control, which refers to an individual’s perception of being able to perform the intended behavior. It is important to note that perceived behavioral control refers not only to believing that one has the necessary skills for the desired behavior, but also to the individual’s perception of what can be done with those skills. TPB posits that the most important factor influencing behavior is intention.

SCT has revealed that entrepreneurial intention and success are greatly influenced by entrepreneurial self-efficacy (Buttner, 2001). Additionally, the literature shows the importance of self-efficacy and emotional intelligence (EI) on intended behavior (McLaughlin, 2019). However, research on EI and entrepreneurial self-efficacy (ESE) is scarce and does not offer clear conclusions in the context of entrepreneurship (Miao et al., 2017a,b; Ingram et al., 2019).

The basic intention-based progress model proposes that the emergence of the entrepreneurial intention process is highly sensitive to initial conditions. Individuals who adopt certain behavioral goals are influenced by external factors and planned behavioral attitudes. External factors include skills, knowledge, and personality traits, among others.

Based on the three aforementioned models, this study tries to deepen the knowledge about the influence of individual variables on entrepreneurial intention. Specifically, the goal of this study was to analyze the relationship among emotional intelligence (EI), entrepreneurial self-efficacy (ESE), and entrepreneurial intention, controlling the effects of individual variables (personality, gender, and age).

This paper is structured as follows: After this introduction, the section “Literature review and research hypotheses” outlines the hypothesized relationships between entrepreneurial intention, emotional intelligences, personality, and entrepreneurial self-efficacy; the “Material and methods” section describes Participants, Measures, Procedures, and Data analyses. “Results” section presents the results of the analyses carried out to examine the relationship between the variables studied, and finally, in the section “Discussion and implications” the limitations and future research, conclusion, and practical implications are presented.

## Literature review and research hypotheses

### Entrepreneurial intention

In order to understand the antecedents of individuals’ behaviors, different models of entrepreneurial intention have been proposed and tested with samples of university students in the years previous to come into the labor market. Social-cognitive theory (Bandura, 1986) and the theory of planned behavior (Ajzen, 2011) are the most important in this context. Intention has been defined as the “indications of a person’s readiness to perform a behavior.” Ajzen (2011, p. 1122). In the entrepreneurial context, Bird describes entrepreneurial intention as “a process, state, or act of conscious willing in the present to make some experience become true, realized, manifested, or created in the future…Thus, intentions can be to do, to be or to have*”* (Bird, 2015, p.143). For this author, it is the state of mind that directs actions toward entrepreneurial behavior. Bird highlights the importance of psychological variables and the impact of people with higher entrepreneurial intention in the development of organizations. Several authors view entrepreneurial intention as the first step and the necessary precursor to entrepreneurial behavior (Kickul et al., 2009; Liñán and Chen, 2009; McLaughlin, 2019).

In psychology, the study of entrepreneurship has mainly focused on examining which individual variables are able to predict entrepreneurial intentions and determining which traits distinguish entrepreneurs from non-entrepreneurs. These studies explore factors affecting motivation to become an entrepreneur, including personal attributes, gender, age, and education (Baron, 2007) as well as the individual’s attitude toward change, competition, monetary rewards, achievement, and autonomy (Delmar and Davidsson, 2000). Relationships between entrepreneurial intention and psychological variables like personality traits (Zhao et al., 2010; Obschonka and Stuetzer, 2017) entrepreneurial self-efficacy (McGee et al., 2009; Murugesan and Jayavelu, 2017), or emotional intelligence (Zampetakis et al., 2009; Ingram et al., 2019) had been also studied.

### Personality traits

There is a substantial body of literature exploring what personality traits influence entrepreneurial intention, mostly under the Big Five personality model (Chao-Tung et al., 2015). In a systematic review on entrepreneurial intentions, Liñán and Fayolle (2015) found that nearly a half of the papers about individual variables and entrepreneurial intention focus on personality.

In the review by Omorede et al. (2015), 39% of the research was designed to study personality in this field, focusing on both general (Zhao et al., 2010; Brandstätter, 2011), and specific personality traits (Rauch and Frese, 2007a,b; Muñiz et al., 2014).

Research reveals that higher scores in extroversion, conscientiousness, and openness and lower in agreeableness and neuroticism are positively associated with entrepreneurial intention (Zhao et al., 2010; López-Núñez et al., 2020).

Extraversion describes a person who is active, is energetic, and enjoys participating in groups. Extraversion is a reliable predictor of good interpersonal relationships and constructive social interactions (Rothmann and Coetzer, 2003). The study by Lee and Tsang (2001) with novel entrepreneurs found that extraversion led to set up communication networks that facilitated their business progress.

Openness to experience refers to a sense of curiosity, open-mindedness, and acceptance of novel experiences (McCrae and Costa, 2003) and is considered an important factor in entrepreneurs, because it is involved in recognizing entrepreneurial opportunities (Zhao and Seibert, 2006; Antoncic et al., 2015). People higher in openness are someone being free to new ideas and ready and receptive to perceive an opportunity, essential to start an entrepreneurial process (Baron, 2007).

Conscientiousness is manifested in goal orientation (the quality of being hardworking and persistent), dependability (the quality of being responsible and careful), and orderliness (being organized and planned; Rothmann and Coetzer, 2003). Conscientious people tend to be efficient, careful, organized, and practical. Studies on entrepreneurship find that conscientiousness is positively related to the long-term survival of a business and to motivation to achieve goals (Singh and deNoble, 2003; Chao-Tung et al., 2015).

Despite the positive aspects of agreeableness, some authors have pointed out its dark side in relation to entrepreneurs (Antoncic et al., 2015). Since in the business environment, relationships can often be adversarial, altruistic behavior may not be a beneficial trait. In this sense, several studies have found that entrepreneurs are lower in agreeableness than non-entrepreneurs (Zhao and Seibert, 2006; Chao-Tung et al., 2015).

Studies suggest that entrepreneurship is positively related to low neuroticism and high emotional stability scores. High levels of anxiety and negative moods, such as anger, are likely to interfere with the ability to make good decisions. People with low emotional stability scores are less likely to deal with problems and stress through positive thinking and direct action. People with high levels of emotional stability carry themselves calmly and confidently and focus on the tasks at hand, even under stress (Zhao et al., 2010).

Although it seems clear that personality is an important antecedent of entrepreneurial intention and entrepreneurship, it is not enough to explain the role of individual variables in the entrepreneurial process. In fact, some authors have indicated that other variables, such as emotional intelligence, can also be significant in predicting entrepreneurial intention and behavior (Andrei et al., 2016; Miao et al., 2018).

The below additional hypotheses are postulated based on these arguments:

*H1*: Personality traits are associated with entrepreneurial self-efficacy.

*H1a*: Neuroticism and agreeableness are negatively related to entrepreneurial self-efficacy.

*H1b*: Extroversion, openness, and conscientiousness show a positive relationship with entrepreneurial self-efficacy.

### Emotional intelligence

The literature on entrepreneurship also highlights the role that emotions play in recognizing opportunities (Foo, 2011; Wincent and Örtqvist, 2011). Emotional intelligence (EI) is the ability to recognize, understand, and handle the emotions (Mayer and Salovey, 1997).

Being an entrepreneur involves making decisions in uncertain and high-risk circumstances where emotions surface due to demands, time pressure, and stress. In addition, to achieve their goals, the entrepreneur is required to be able to properly regulate emotions in social interactions. Therefore, entrepreneurship is a highly emotional work context, which requires the regulation of emotions to display them appropriately to a variety of stakeholders. Research on EI is relevant in psychology, both in clinical and applied psychology (Petrides et al., 2016). In general, people with high EI show a higher stress tolerance and better use their emotional regulation skills. In addition, self-perceived emotions tend to have greater creativity and proactivity, which influences entrepreneurial behavior (Ingram et al., 2019). EI is related to successful decision-making and greater satisfaction with life (Bastian et al., 2005). People with higher EI scores are more imaginative, are proactive, and show more entrepreneurial intention than those with lower scores (Cross and Travaglione, 2003). People with high emotional intelligence show a higher stress tolerance and better use their emotional regulation skills.

Zampetakis et al. (2009) argue that EI affects entrepreneurial behavior in two ways: The first is through the self-evaluation of emotional efficacy (workers with high EI may show high tolerance to stress); and the second refers to the fact that individuals with high EI tend to have higher affectivity, related to proactivity and creativity, thus facilitating entrepreneurial behavior. They studied the relationship between entrepreneurial behavior and emotional intelligence and found that there is a direct effect of EI on entrepreneurial behavior.

In the work context, research has focused mainly on the role of EI in performance, engagement, job effectiveness, health, and job satisfaction (Miao et al., 2016, 2017a,b), and less attention has been given to its role as an antecedent of entrepreneurial intentions and its relationship with other individual variables, such as self-efficacy (McLaughlin, 2019). Entrepreneurial activity requires establishing interpersonal relationships, which involves building trust, establishing networks, and managing adversity. All this must be done in an environment of high uncertainty, which strengthens the role of emotion management.

The literature highlights the key role of emotional intelligence in entrepreneurial intention and its relationship with other individual variables in both student and entrepreneur samples (Ahmetoglu et al., 2011; Miao et al., 2018). In a study with college students, Mortan et al. (2014) found that emotional intelligence positively affects self-efficacy and that this mediates the relationship between emotional intelligence and entrepreneurial intention. In another study with a sample of 943 students enrolled in management courses, Ingram et al. (2019) demonstrated that interpersonal skills, which involve recognizing and managing emotions, have a positive effect on entrepreneurship.

Therefore, this study postulates the below hypothesis:

*H2*: Emotional intelligence dimensions are positively associated with entrepreneurial self-efficacy.

### Self-efficacy

Self-efficacy is defined as “…belief in one’s capabilities to mobilize the motivations, cognitive resources, and courses of action needed to meet given situational demands…” (Wood and Bandura, 1989, p. 364). This motivational construct has been applied to the field of entrepreneurship, giving rise to the concept of entrepreneurial self-efficacy (ESE).

In this context, ESE refers to the confidence that an individual has of his or her capacity to accomplish the entrepreneurial process (Chen et al., 1998). People with high ESE show confidence in their own abilities to achieve their goals in entrepreneurial areas, set challenging goals, show perseverance, and recover quickly from failure. ESE is a relevant antecedent of venture performance (Miao et al., 2017a,b).

Self-efficacy beliefs affect a person’s expectations, goals, and decisions. It can be improved through experience, so learning plays an important role in its development (Bandura et al., 2001). People with high levels of self-efficacy make more effort to comply with their commitments and associate failure with internal factors, rather than external factors (Hechavarria et al., 2012).

Research focused on the development of ESE considers variables, such as experience, vicarious learning, and social persuasion using social cognitive theory (Bandura, 1977) as a model. Research shows a relationship between ESE and entrepreneurial intention (Barbosa et al., 2007; Kickul et al., 2009). With a sample of college students, Hu and Ye (2017) provided empirical evidence that ESE is a key cognitive predictor of entrepreneurial intention.

The knowledge base and capabilities that can be developed through experience or higher education programs are considered to have a positive effect on an individual’s motivation and self-efficacy for entrepreneurship. Newman et al. (2019) showed that entrepreneurial intention is the most widely studied outcome of ESE. In the field of higher education, the positive relationship between ESE and entrepreneurial intention has also been demonstrated (Piperopoulos and Dimov, 2015; Hu and Ye, 2017). These results can be used to help ensure that entrepreneurial education is more effective in educational programs, professional training, and vocational guidance.

The results on the relationship of the ESE with entrepreneurship have increased interest in knowing its mediating influence on entrepreneurial intention. Kumar and Shukla (2019) explored the role of ESE as mediating the effect of proactivity and creativity on entrepreneurial intention in a sample of 484 management students. They found that ESE was the strongest predictor of entrepreneurial intention. In another study, Prabhu et al. (2012) analyzed the role of ESE in mediating the influence of personality on entrepreneurship and found that ESE had a robust effect on the correlation between personality and entrepreneurship.

ESE has been emphasized as a key antecedent of entrepreneurial intentions. Individuals are more commonly inclined to choose situations in which they anticipate more personal control and to avoid situations in which they anticipate less personal control. Entrepreneurial self-efficacy progresses over time and is influenced by internal and external factors, such as education, economic context, and psychological variables (Miao et al., 2017a,b).

Despite the research that demonstrates the important role played by EI and ESS as antecedents of entrepreneurial intention (McLaughlin, 2019), few studies have addressed the relationship between both variables in the entrepreneurial process, and these have focused on vocational college students (Newman et al., 2019; Wen et al., 2020).

The following hypotheses are proposed to examine the possible effect of ESS:

*H3*: Entrepreneurial self-efficacy is positively associated with entrepreneurial intention.

*H4*: Entrepreneurial self-efficacy mediates the relationship between emotional intelligence and entrepreneurial intention.

## Materials and methods

### Participants

A non-experimental, cross-sectional design was used in this research. Non-probabilistic sampling was used. The participants were 1,593 college students, aged between 17 and 69 (*M* = 21.0, *SD* = 3.80). Data were provided from several disciplines, such as humanities (4.7%), social sciences (40.1%), experimental sciences (6.0%), and health sciences (49.2%). Women made up a majority (68.2%) of the sample. Most participants (78.0%) were studying, and 22.0% were both studying and working.

### Measures

#### Entrepreneurial intention

It was evaluated through a Likert-type scale with six items (Liñán and Chen, 2009) that assess behavioral intention in one factor. The items ask about the degree of agreement in a range of seven points. The items on this scale are like “My professional goal is to become an entrepreneur” or “I am determined to create a firm in the future.” The higher the score on the scale, the higher the level of entrepreneurial intention. The reliability (internal consistency) of this scale with our sample was high (Cronbach’s alpha = 0.93).

#### Entrepreneurial self-efficacy

The Perceived Behavioral Control Scale by Liñán and Chen (2009) was used to assess ESE. It includes six items that ask for the degree of agreement in a seven-point Likert scale. Items are like “To start a firm and keep it working would be easy for me” or “If I tried to start a firm, I would have a high probability of succeeding.” In this study, Cronbach’s alpha was 0.90.

#### Emotional intelligence

It was evaluated with the Spanish Modified Version of the Trait Meta-Mood (TMMS-24; Fernández-Berrocal et al., 2004). This instrument has 24 items which assess three emotional intelligence dimensions: emotional attention, emotional clarity, and emotional repair. Cronbach’s alpha was 0.89 for emotional attention, 0.87 for emotional clarity, and 0.85 for emotional repair.

### Control variables

According to revised research, factors such as gender and age have an impact on entrepreneurial intention (Zisser et al., 2019; Pandang et al., 2022). The gender was assessed as male and female. The first options were coded as “0,” and the second options were coded as “1.”

#### Personality

The Spanish version (Cordero et al., 1999) of the NEO-Five Factor Inventory (NEO-FFI; Cordero et al., 1999) was used. This instrument consists of 60 items that evaluate five factors: neuroticism (N), extraversion (E), openness to experience (O), agreeableness (A), and conscientiousness(C). Adequate reliability was obtained with our participants: Cronbach’s alpha (N) = 0.83; Cronbach’s alpha (E) = 0.85; Cronbach’s alpha (O) = 0.82; Cronbach’s alpha (A) = 0.71; Cronbach’s alpha (C) = 0.80.

### Procedure

The participants answered the questionnaires in the paper-and-pencil format in a single session of about 45 min. At the beginning of the session, the researchers explained the instructions and the guarantees regarding anonymity and confidentiality of the data. All participants signed a “consent to participate.” The research was approved by the Ethics Commission of the Faculty of Psychology of the Complutense University of Madrid.

### Data analyses

We use SPSS 25.0 for all statistical analyses. First, the mean, standard deviation, and correlations for all the variables included in the study were calculated.

To examine whether EI dimensions would explain the incremental variation in ESE that mediates the intention to become an entrepreneur, beyond the level attributable to personality traits and demographic variables, we performed two multiple hierarchical regression analyses. The indirect mediation role of ESE was analyzed using the procedure for testing multiple mediations described by Mac Kinnon (2008), which consists of estimating two separate regression equations. The basic strategy consists of a three-step hierarchical regression: Demographic variables are entered as covariates in the first step, the Big Five personality factors are added in the second step to control for any possible influence of this measure on ESE, and the three dimensions of the EI are entered in the last step. A similar procedure is also repeated for the second four-step multiple regression analysis, adding ESE as a mediator in the final step. Hierarchical regression is a subset of regression methods that attempt to generate theory-driven evidence for a given effect. In hierarchical regression, predictor variables are entered into the model in pre-determined iterations to see how the change in R^2^ is affected. The hierarchical regression analysis occurs in iterations. The first iteration will be with the most highly correlated variable to the outcome, and then subsequently add in other variables that have some association on the outcome. If the entry of a variable leads to a significant increase in R^2^ as per the F-statistic, then evidence of its predictive ability can be noted, as R^2^ shows what proportion of the variation in the dependent variable is accounted for by the model. This same analysis procedure has been applied in many studies within the field of psychology, and specifically around entrepreneurial intention, an example is found in Mortan et al. (2014).

## Results

### Preliminary analyses

Table 1 shows the correlation coefficient matrix and descriptive statistics. The internal reliabilities of each measure (Cronbach’s alphas) are in brackets.

**Table 1:** Means, standard deviations (SD), correlations, and reliabilities (on the diagonal in brackets).

	M	SD	1	2	3	4	5	6	7	8	9	10
1. Emotional attention	28.22	6.42	(0.89)									
2. Emotional clarity	27.36	5.84	0.23^**^	(0.87)								
3. Emotional repair	27.71	6.13	0.11^**^	0.39^**^	(0.85)							
4. Entrepreneurial intention	3.72	1.48	0.03	0.13^**^	0.20^**^	(0.93)						
5. Entrepreneurial self-efficacy	3.16	1.30	0.04	0.21^**^	0.27^**^	0.60^**^	(0.90)					
6. Neuroticism	22.77	8.09	0.30^**^	−0.31^**^	−0.43^**^	−0.14^**^	−0.23^**^	(0.83)				
7. Extraversion	30.87	7.79	0.11^**^	0.24^**^	0.42^**^	0.19^**^	0.23^**^	−0.35^**^	(0.85)			
8. Openness	27.97	7.02	0.31^**^	0.12^**^	0.19^**^	−0.05	0.01	0.09^**^	0.16^**^	(0.82)		
9. Agreeableness	28.87	6.40	0.18^**^	0.12^**^	0.30^**^	0.02	−0.02	−0.17^**^	0.30^**^	0.19^**^	(0.71)	
10. Conscientiousness	30.57	7.22	0.06^*^	0.20^**^	0.22^**^	0.11^**^	0.14^**^	−0.24^**^	0.16^**^	0.05	0.13^**^	(0.80)

* *p* < 0.05 and

** *p* < 0.01.

### Hierarchical regression analysis

Table 2 shows the results of the hierarchical regression analysis to predict ESE. The results show that the variables explain 15.3% of the total variance in the model (*R^2^ = 0*.15, *p* < 0.01). Age and gender explain 3.9%, personality traits explain 8.7%, and emotional intelligence dimensions account for 2.7%. The three types of variables studied in the hierarchical model, demographic (gender and age), personality traits, and emotional intelligence, are related to ESE. Results indicate that gender shows a negative relation with ESE (*β* = −0.149, *p* < 0.001) suggesting that women have a lower ESE than men. Age shows a positive relation with ESE (*β* = 0.114, *p* < 0.001).

**Table 2:** Results of hierarchical regression analysis to predict entrepreneurial self-efficacy based on age, gender, personality, and emotional intelligence.

Variables	β	*R^2^*	Δ*R*^2^	Δ*F*	Sig.
Step 1		0.039	0.039	32.14	0.000
Age	0.114^***^				
Gender (0 = men; 1 = women)	−0.149^***^				
Step 2		0.126	0.087	31.66	0.000
Neuroticism	−0.112^***^				
Extraversion	0.211^***^				
Openness	0.011				
Agreeableness	−0.094^***^				
Conscientiousness	0.101^***^				
Step 3		0.153	0.027	16.92	0.000
Emotional attention	0.076^**^				
Emotional clarity	0.056^*^				
Emotional repair	0.139^***^				

β are the standardized regression coefficients.

* *p* < 0.05;

** *p* < 0.01;

*** *p* < 0.001.

The hypotheses 1, 1a, and 1b have been partially confirmed, since all personality traits, except openness, show a relationship with entrepreneurial self-efficacy. In spite of this, the relationships between the dimensions of personality and ESE fulfill the hypotheses proposed. While extraversion (*β* = 0.211, *p* < 0.001) and conscientiousness (*β* = 0.101, *p* < 0.001) show a positive relation, neuroticism (*β* = −0.112, *p* < 0.001) and agreeableness (*β* = −0.094, *p* < 0.001) show a negative relation with entrepreneurial self-efficacy.

Hypothesis 2 was also confirmed by obtaining a significant positive relationship between EI and ESE: emotional attention (*β* = 0.076, *p* < 0.01), emotional clarity (*β* = 0.056, *p* < 0.05), and emotional repair (*β* = 0.139, *p* < 0.001).

Finally, a second multiple regression analysis was performed with four steps to which ESE was added (Table 3). Age and gender account for 1.5% of the variance. Personality traits explain 5.5%, and emotional intelligence dimensions explain 1.6%. ESE shows a positive relationship with entrepreneurial intention (*β* = 0.585, *p* < 0.001). Overall results shown that this model accounts for 37.4% of the total variance, so the hypotheses three and four were confirmed.

**Table 3:** Results of hierarchical regression analysis for age, gender, personality, and emotional intelligence predicting entrepreneurial intention mediated by ESE.

Variables	β	R^2^	Δ*R*^2^	Δ*F*	Sig.
Step 1		0.015	0.015	12.08	0.000
Age	0.100^***^				
Gender (0 = men; 1 = women)	−0.060^*^				
Step 2		0.070	0.055	18.65	0.000
Neuroticism	−0.027				
Extraversion	0.202^***^				
Openness	−0.067^**^				
Agreeableness	−0.035				
Conscientiousness	0.078^**^				
Step 3		0.086	0.016	9.37	0.000
Emotional attention	0.052				
Emotional clarity	0.019				
Emotional repair	0.128^***^				
Step 4		0.376	0.290	734.47	0.000
Entrepreneurial self-efficacy	0.585^***^				

β are the standardized regression coefficients.

* *p* < 0.05;

** *p* < 0.01;

*** *p* < 0.001.

**Figure 1:**
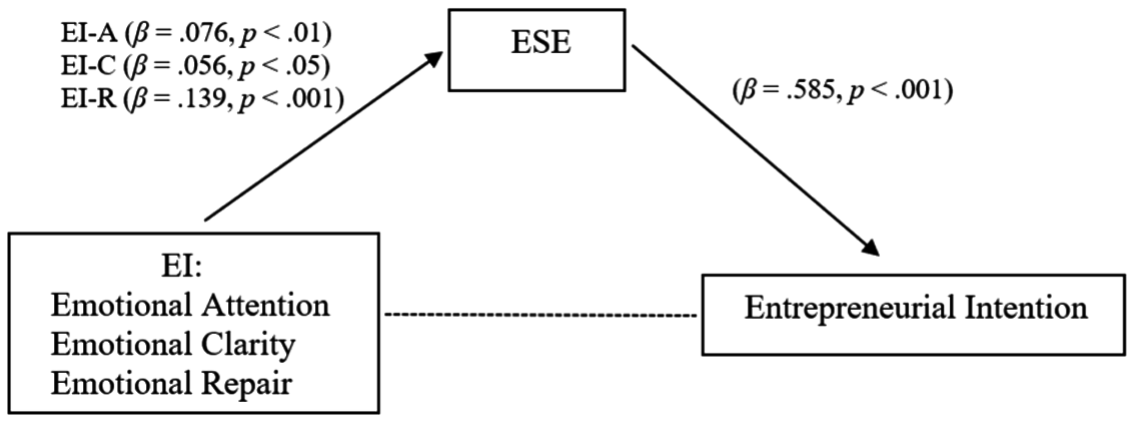
Final model.

In summary, the results revealed a positive and significant relationship between ESE and the intention to become an entrepreneur impacted by the role of EI. Figure 1 represents the relations between ESE, EI and entrepreneurial intention, in the final model obtained controlling the effects of gender, age, and personality.

## Discussion and implications

### Discussion

The main objective of this paper was to analyze the relationship among EI, ESE, and entrepreneurial intention, controlling for the effects of the individual variables: personality and demographic variables (age and gender). The results confirmed all the proposed hypotheses: The personality traits are associated with entrepreneurial self-efficacy, emotional intelligence (EI) has a positive influence on entrepreneurial intention, and ESE mediates this relationship. This is inconsistent with some previous studies (Ferreira et al., 2022; Fu et al., 2022). All the variables studied—control variables (gender, age and personality traits) and emotional intelligence—are related to entrepreneurial self-efficacy.

### Demographic variables

About gender, some theories suggest that men are expected to undertake more ventures than women (Bar Nir et al., 2011). In this sense, studies explored the impact of gender on motivations to become entrepreneurs from the point of view of self-efficacy. Our results agree with those found by other authors (Díaz-García and Jiménez-Moreno, 2010; Dempsey and Jennings, 2014). In general, women have a lower level of ESE than men. Zampetakis et al. (2017) found that women tend to show a lack of confidence in their ability to engage in entrepreneurial behaviors. Although many studies find these same results, including studies by the Global Entrepreneurship Monitor (GEM; Wennberg et al., 2013), others suggest that the difference between men and women is not significant (Coleman and Kariv, 2014). Zhao et al. (2005) analyzed graduate students and found no significant role of gender in entrepreneurial self-efficacy.

In general, research shows that women showed less entrepreneurial intention than men (Strawser et al., 2021; Serrano-Pascual and Carretero-García, 2022). Some authors postulate that the differences in the results on the effect of gender on entrepreneurial self-efficacy may be influenced by personal attitudes toward entrepreneurship (Leroy et al., 2009; Baluku et al., 2020) or by gender role stereotypes (Sweida and Woods, 2015). Zisser et al. (2019), found that women and men varied in personality dimensions related to self-esteem, energy, risk attraction, and ambition; however, when women and men with high levels of EI are compared, they showed similar personality dispositions. Other research indicates that differences in ESE are associated with areas of specialization traditionally considered as consistent with gender stereotypes (Pandang et al., 2022). Regarding age, the older the participant, the greater their perception of entrepreneurial self-efficacy.

### Personality

The first conclusion is that openness turned out to be unrelated to ESE. Extroversion and conscientiousness had a positive association with ESE, while the relation between ESE and neuroticism and agreeableness was negative (the higher the score in these latter traits, the lesser the entrepreneurial self-efficacy). These results are partially maintained in the second analysis, which evaluated predictors of entrepreneurial intention mediated by self-efficacy. In this case, extroversion and conscientiousness continued to positively predict entrepreneurial intention. Openness was negatively correlated with entrepreneurial intention, which indicated that the greater one’s openness, the lower their entrepreneurial intention; finally, agreeableness and neuroticism were not significant predictors of entrepreneurial intention.

In general, these results agree with those found in the literature, in which extroversion and conscientiousness were associated with entrepreneurial intention, while agreeableness and neuroticism were negatively associated (Zhao et al., 2010; Brandstätter, 2011; Antoncic et al., 2015). The negative relationship between openness and entrepreneurship is striking, given that prior studies found high correlations between the two (Zhao et al., 2010; Antoncic et al., 2015). However, our results agree with those found by Mei et al. (2017). Future research should consider that there are multiple configurations of Big Five personality traits that vary by business form, environment, and type of entrepreneur (Şahin et al., 2019; Salmony and Kanbach, 2021).

### Emotional intelligence

All dimensions of EI had a significant, positive correlation with ESE and entrepreneurial intention, especially emotional repair. These results match with those found by other authors (McLaughlin, 2019; Wen et al., 2020). As indicated above, there are few previous studies that have examined the influence of EI on entrepreneurial intention mediated by ESE. Our work expands knowledge of this relationship and found similar results to the obtained by other researchers regarding the moderating role of ESE in entrepreneurial intention (Newman et al., 2019; Huezo-Ponce et al., 2021; Wu and Tian, 2022).

The finding of our study suggests that people with higher scores in EI also have greater ESE. Managing and regulating one’s own emotions and the emotions of others is an essential skill for the entrepreneurial process (Sadri et al., 2011; Ramoglou and Tsang, 2016). In a context where decision-making is recurrent, characterized by ambiguity and uncertainty, to have the ability to control and manage one’s emotions, together with a high perception of self-confidence and self-efficacy, it will allow people to recognize opportunities, manage interpersonal relationships more efficiently, and have a higher tolerance for risk and uncertainty (Hirsh et al., 2012; Davidsson, 2015).

## Theoretical implications

First, this study investigated the effects of individual variables on entrepreneurial self-efficacy related to EI and entrepreneurial intention. The results confirm previous studies (Mortan et al., 2014; Chien-Chi et al., 2020) and extend other findings on the role of entrepreneurial self-efficacy in the relationship between emotional intelligence and entrepreneurial intention using sociodemographic variables and personality traits as control variables which have not been sufficiently studied so far.

Second, the results on the relationship between personality traits and entrepreneurial intention stand out. In general, the results confirm previous studies but there are some contradictory findings. We found that openness was not directly related to entrepreneurial intention (Mei et al., 2017) contradicting the findings of others research (Kerr et al., 2018). The reason could be that the participants with high openness scores were university students who have a wide range of interests during this period, which limits their entrepreneurial possibilities.

These findings contribute to research on the influence of personality traits on entrepreneurial intention and broaden the discussion on the role of openness in entrepreneurial intention in university students.

## Practical implications

Our results may have practical implications for the design of training strategies aimed at fostering entrepreneurial initiative. Most entrepreneurship education, both to encourage entrepreneurial initiative as well as training designed for young entrepreneurs, is focused on technical planning and management knowledge, overlooking individual skills such as those highlighted in this study. The main conclusion that we obtain from this study is that the intention to start a business depends to a great extent on ESE; therefore, every entrepreneurship training and promotion program must include activities aimed at increasing ESE. The results also show that ESE is associated with EI, and especially with the Emotional Repair dimension. In stressful situations, very frequent during the entrepreneurial process, we can think of emotional attention, clarity, and repair as steps that we have to follow. First, we need to pay attention to what we are feeling, second, we need clarity about the emotion, and third, we need a strategy to repair the emotion, but entrepreneurial training should focus on the last: finding an effective strategy for repair and to better control and manage their own emotions. In addition, training programs need to implement a gender-sensitive approach, since women seem to have less ESE and therefore less entrepreneurial intention than men. One way could be including activities that facilitate the exploration of women’s motivations and aspirations, identification and understanding of emotions, as well as self-regulation of emotions, since women tend to suffer more stress, often due to difficulties in reconciling work and personal life, which makes it difficult for them to succeed in their business actions.

We believe that our results can be especially valuable for educational institutions that wish to provide education for entrepreneurship, as well as organizations that want to develop internal talent through intrapreneurship actions.

## Limitations and future research

The present work shows evidence for the relationship between EI and entrepreneurial intention and the mediating role of ESE. However, it has some limitations which should be considered in future research. First, a longitudinal study would be appropriate to investigate whether the intention translates into action. Second, although the sample is made up of students from all fields of knowledge, it would be ideal to expand the sample from the humanities and experimental sciences fields, and to analyze differences between the groups. Traditionally, research on entrepreneurial intention has been performed in the academic fields of business and enterprise, but it would be good to broaden the scope to all other academic areas. Third, this study was limited to a specific geographical area, and it would be interesting to carry out similar studies in other countries and different cultures. Few previous studies were found which address the relationship among EI, ESE, and entrepreneurial intention, so new studies should be conducted to provide more evidence about the relationship between these variables.

Finally, we must point out that, except for the association between ESE and entrepreneurial intention, some of the relationships found cannot be considered high (Tables 2, 3), although they are statistically significant. In this sense, it is evident that factors other than those considered here also influence the entrepreneurial intention and the ESE (socioeconomic level, social context, market, etc.).

Entrepreneurship is closely related to social and economic factors, and family is one of the main factors influencing university students to start their own entrepreneurship project (Antoncic et al., 2021). Therefore, it would be very interesting to include these additional variables in future research to examine their effect on entrepreneurial behavior as marketing self-efficacy (Antoncic et al., 2016).

## Conclusion

This research focused on the study of one part of the entrepreneurial process, specifically, on analyzing individual variables that are antecedents of entrepreneurial intention and their relationships.

The results reveal that the classic profile of the Big Five associated with entrepreneurial behavior, characterized by high scores in extroversion and conscientiousness and low scores in neuroticism and agreeableness, shows high self-efficacy and entrepreneurial intention. Given the level of complexity and uncertainty that the entrepreneurial process implies, there is increasing evidence about the importance of emotions in understanding entrepreneurial behavior. People with high emotional intelligence show more capacity to identify and handle emotions and they show higher levels of self-efficacy and confidence to take on these challenges.

## Data availability statement

The raw data supporting the conclusions of this article will be made available by the authors, without undue reservation.

## Ethics statement

The studies involving human participants were reviewed and approved by Deontology Commission of the Faculty of Psychology of the Complutense University of Madrid, obtaining a favorable report on October 2020 (Ref. 2020/21–005). The patients/ participants provided their written informed consent to participate in this study.

## Author contributions

MIL-N, SR-V, and ED-R contributed to the conception and design of the study and revised the final version of the manuscript. MIL-N organized the database and wrote the first draft of the manuscript. SR-V performed the statistical analysis. All authors contributed to the article and approved the submitted version.

## Conflict of interest

The authors declare that the research was conducted in the absence of any commercial or financial relationships that could be construed as a potential conflict of interest.

## Publisher’s note

All claims expressed in this article are solely those of the authors and do not necessarily represent those of their affiliated organizations, or those of the publisher, the editors and the reviewers. Any product that may be evaluated in this article, or claim that may be made by its manufacturer, is not guaranteed or endorsed by the publisher.
