# Supplementary material for: The role of individual variables as antecedents of entrepreneurship processes: Emotional intelligence and self-efficacy
Source: Front Psychol. 2022 Oct 25;13:978313. doi: 10.3389/fpsyg.2022.978313 (PMC9671165; doi:10.3389/fpsyg.2022.978313)
Supplement: Supplementary file 1 [file Data_Sheet_1.PDF]

Supplementary material

Table 1. Items used to measure research constructs.

**1. Entrepreneurial Intention**<br><br>1.1. I am ready to do anything to be an entrepreneur.<br>1.2. My professional goal is to be an entrepreneur.<br>1.3. I will make every effort to start and run my own business.<br>1.4. I am determined to create a business venture in the future.<br>1.5. I have very seriously thought in starting a firm.<br>1.6. I have the firm intention to start a firm some day.	(Liñán and Chen, 2009)
**2. Entrepreneurial Self-Efficacy**<br><br>2.1. To start a firm and keep it working would be easy for me<br>2.2. I am prepared to start a viable firm<br>2.3. I can control the creation process of a new firm<br>2.4. I know the necessary practical details to start a firm<br>2.5. I know how to develop an entrepreneurial project<br>2.6. If I tried to start a firm, I would have a high probability of succeeding	(Liñán and Chen, 2009)
**3. Personality**<br><br>3.1. I often feel inferior to others<br>…<br>3.46. I am seldom sad or depressed<br>…<br>3.60.	Spanish version (Cordero et al., 1999) of the NEO-Five Factor Inventory (NEO-FFI; Costa and McCrae, 1999)
**4. Emotional Intelligence**<br><br>4.1. I pay a lot of attention to my feelings<br>…<br>4.24. When I am angry, I try to change my state of mind	Spanish Modified Version of the Trait Meta-Mood (TMMS-24) (Fernandez-Berrocal et al., 2004).
